# How do children with severe underweight and wasting respond to treatment? A pooled secondary data analysis to inform future intervention studies

**DOI:** 10.1111/mcn.13434

**Published:** 2022-10-19

**Authors:** Gloria A. Odei Obeng‐Amoako, Heather Stobaugh, Stephanie V. Wrottesley, Tanya Khara, Paul Binns, Indi Trehan, Robert E. Black, Patrick Webb, Martha Mwangome, Jeanette Bailey, Paluku Bahwere, Carmel Dolan, Erin Boyd, André Briend, Mark A. Myatt, Natasha Lelijveld

**Affiliations:** ^1^ Clinical Epidemiology Unit, School of Medicine, College of Health Sciences Makerere University Kampala Uganda; ^2^ Action Against Hunger USA New York City New York USA; ^3^ Friedman School of Nutrition Science and Policy at Tufts University Boston Massachusetts USA; ^4^ Emergency Nutrition Network (ENN) Kidlington UK; ^5^ Action Against Hunger UK London UK; ^6^ Departments of Paediatrics, Global Health, and Epidemiology University of Washington Seattle Washington USA; ^7^ Institute for International Programmes Johns Hopkins Bloomberg School of Public Health Baltimore Maryland USA; ^8^ Kenya Medical Research Institute (KEMRI) Centre for Geographic Medicine Research‐Coast Kilifi Kenya; ^9^ International Rescue Committee New York City New York USA; ^10^ Center for Epidémiology, Biostatistics and Clinical Research (CR2), School of Public Health Université Libre de Bruxelles Brussels Belgium; ^11^ N4D London UK; ^12^ USAID/BHA Washington District of Columbia USA; ^13^ Department of Nutrition, Exercise and Sports, Faculty of Science University of Copenhagen Frederiksberg Denmark; ^14^ Center for Child Health Research, Faculty of Medicine and Health Technology Tampere University Tampere Finland; ^15^ Brixton Health Llwyngwril Gwynedd Wales UK

**Keywords:** anthropometry, child nutrition, malnutrition, stunting, underweight, wasting

## Abstract

Children with weight‐for‐age z‐score (WAZ) <−3 have a high risk of death, yet this indicator is not widely used in nutrition treatment programming. This pooled secondary data analysis of children aged 6–59 months aimed to examine the prevalence, treatment outcomes, and growth trajectories of children with WAZ <−3 versus children with WAZ ≥−3 receiving outpatient treatment for wasting and/or nutritional oedema, to inform future protocols. Binary treatment outcomes between WAZ <−3 and WAZ ≥−3 admissions were compared using logistic regression. Recovery was defined as attaining mid‐upper‐arm circumference ≥12.5 cm and weight‐for‐height z‐score ≥−2, without oedema, within a period of 17 weeks of admission. Data from 24,829 children from 9 countries drawn from 13 datasets were included. 55% of wasted children had WAZ <−3. Children admitted with WAZ <−3 compared to those with WAZ ≥−3 had lower recovery rates (28.3% vs. 48.7%), higher risk of death (1.8% vs. 0.7%), and higher risk of transfer to inpatient care (6.2% vs. 3.8%). Growth trajectories showed that children with WAZ <−3 had markedly lower anthropometry at the start and end of care, however, their patterns of anthropometric gains were very similar to those with WAZ ≥−3. If moderately wasted children with WAZ <−3 were treated in therapeutic programmes alongside severely wasted children, we estimate caseloads would increase by 32%. Our findings suggest that wasted children with WAZ <−3 are an especially vulnerable group and those with moderate wasting and WAZ <−3 likely require a higher intensity of nutritional support than is currently recommended. Longer or improved treatment may be necessary, and the timeline and definition of recovery likely need review.

## Key messages

- Children with WAZ <−3 have a high risk of death, yet this indicator is not widely used in nutrition treatment programming.
- WAZ <−3 is common among both severely and moderately wasted children (64% and 43%, respectively), and those with WAZ <−3 do not meet conventional definitions of nutritional recovery as often as other wasted children. While they do gain weight in a similar pattern as other wasted children, they have further to catch up.
- Children with wasting and WAZ <−3 likely require longer or more tailored treatment, especially children with both moderate wasting and WAZ <−3, who are usually not eligible for therapeutic feeding.
- An intervention trial is needed to further explore these hypotheses and must include non‐wasted children with WAZ <−3, who are currently not represented in this secondary data.

## Graphical abstract

Children with weight‐for‐age z‐score (WAZ) <−3 have a high risk of death, yet this indicator is not widely used in nutrition treatment programming. We found that WAZ <−3 is common among both severely and moderately wasted children (64% and 43%, respectively), and those with WAZ <−3 do not meet conventional definitions of nutritional recovery as often as other wasted children. While they do gain weight in a similar pattern as other wasted children, they have further to catch up. Children with wasting and WAZ <−3 likely require longer or more tailored treatment.

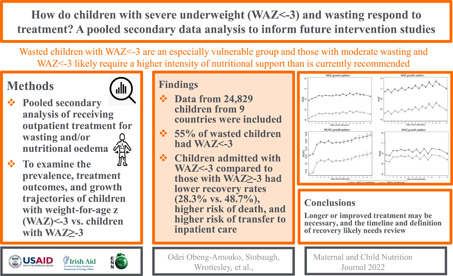

## 1 INTRODUCTION

Undernutrition in children under 5 years of age remains persistently high globally, with the COVID‐19 pandemic further exacerbating this burden across low‐and middle‐income countries (Osendarp et al., 2021). Undernutrition in early life, including manifestations such as stunting (low height‐for‐age), wasting (low weight‐for‐height and/or mid‐upper arm circumference [MUAC]), bilateral pitting oedema, and underweight (low weight‐for‐age), is associated with significantly higher rates of morbidity and mortality in childhood (McDonald et al., 2013). It also has long‐term implications for educational achievement, economic productivity and the risk of non‐communicable disease in later life (Black et al., 2013; Murray et al., 2020).

Although stunting and wasting share common drivers, consequences and causal pathways, often occurring concurrently in the same children, they continue to be targeted separately in nutrition policy and programming (Khara et al., 2018; Thurstans et al., 2021). Stunting and wasting may already be present at birth and the incidence of both forms of undernutrition peaks within the first 6 months of life (Christian et al., 2013; Victora et al., 2021). Experiencing an episode of wasting in early life leads to an increased risk of further wasting in later life (Mertens et al., 2020). Previous research has shown that children with multiple anthropometric deficits, including concurrent wasting and stunting (WaSt) are 12 times more likely to die than those with no anthropometric deficit compared to a twofold increase in those with wasting only (McDonald et al., 2013). This highlights the need to re‐examine the extent to which nutrition and health programming are targeting the highest‐risk children for treatment (Bailey et al., 2021; McDonald et al., 2013; Myatt et al., 2018).

Currently, MUAC <11.5 cm, weight‐for‐height z‐score (WHZ) <−3, and the presence of oedema are used as independent criteria for admission of children 6–59 months into therapeutic feeding programmes. These criteria fail to capture children who are moderately wasted and concurrently stunted, despite the fact that their near‐term mortality risk is similar to that of severely wasted children (Myatt et al., 2018). Data indicates that all WaSt children are also underweight, meaning that the weight‐for‐age z‐score (WAZ) can be used to identify WaSt (Thurstans et al., 2021). The most effective combination of indicators for identifying those children at the highest risk of death, including those with WaSt, is WAZ <−3 and MUAC <11.5 (Myatt et al., 2019). Thus, including WAZ <−3 as an additional independent criterion for admission into therapeutic treatment programmes may improve the targeting of malnourished children with the greatest risk of mortality (Bailey et al., 2021; Myatt et al., 2018). However, as low WAZ is not currently used at admission or routinely monitored during wasting treatment, little is known about how this group of children respond to either therapeutic or supplementary treatment protocols.

In this study, we aimed to conduct a pooled secondary data analysis using existing datasets of children treated for wasting, during research studies and high‐quality community‐based management of acute malnutrition (CMAM) operational programmes. The objectives were, in children being treated for wasting and/or nutritional oedema: to (1) examine the prevalence and treatment outcomes of children with WAZ <−3; (2) explore growth trajectories of children with WAZ <−3; and (3), examine how varying levels of treatment affect treatment outcomes and growth trajectories for children with WAZ <−3. These results may help inform future research protocols and operational programmes regarding the potential use of WAZ <−3 as an indicator for admitting children for nutritional care and the appropriate intensity of treatment for this group.

## 2 METHODS

### 2.1 Data acquisition

Datasets included in this study were solicited via an open call for data circulated by Action Against Hunger and the Wasting and Stunting Technical Interest Group (WaSt TIG) coordinated by the Emergency Nutrition Network. To be eligible for inclusion, datasets needed to:

- Include individualised, child‐level data for children aged 6–59 months admitted for outpatient management of moderate or severe wasting (also known as MAM and SAM) (based on criteria of MUAC, oedema, or weight for height);
- Have been collected as part of a rigorous research study or implemented in a high‐quality programme that collected regular monitoring data;
- Include the following variables as a minimum: child age, oedema status, height/length, weight, MUAC, and the date upon admission to treatment and at each follow‐up visit.

Any additional contextual information regarding the type of programme, treatments provided, and morbidity information was also sought. Data were not limited by language or date. All original study protocols and ethical approval documentation from the individual studies were also sought.

### 2.2 Data extraction and cleaning

Anonymised data were compiled into one master dataset. The following variables were extracted from all datasets: intervention type, date, date of birth, age, sex, weight, height/length, MUAC and oedema at admission and date, weight, height/length, MUAC, and nutritional oedema at each follow‐up visit as well as recorded treatment outcomes (death, defaulted, transferred). Children with missing age and sex information were excluded, along with data on children aged <6 and >59 months upon admission to treatment. Children who were not classified as wasted based on current programmatic definitions (i.e., MUAC ≥12.5 or WHZ ≥−2) and did not have nutritional oedema were also excluded. The following additional variables were included where available: morbidity (diarrhoea, respiratory infection/cough, fever, rash, and vomiting) during the treatment period and HIV status at admission. Implausible height/length values (>120 cm or <60 cm) and MUAC values (>20 cm) were censored (i.e., assigned a missing value).

Where the birth dates of children were available, age (months) was calculated. Where birth dates were not available, age (months) from the original dataset was used. In attempts to standardise, the admission status and the treatment outcome variable were generated based on anthropometric measurements, rather than classifications used in the original datasets. Severe wasting without oedema was defined as MUAC <11.5cm or WHZ <−3; moderate wasting was defined as MUAC 11.5–12.4 cm or WHZ −2 to −3, and absence of oedema. Recovery was defined as attaining MUAC ≥12.5 and WHZ ≥−2 and no oedema, within a period of 17 weeks (119 days) of admission (Bailey et al., 2020). These recovery criteria were not necessarily the same as those used in the original studies and therefore are not indicators of programme quality.

Non‐response was defined as still having MUAC <12.5 or WHZ <−2 or oedema after the point of 17 weeks of treatment. Due to varying protocols between treatment programmes, some children continued to receive treatment beyond 17 weeks and were later classified as recovered in the original studies and/or programmes. However, for the purpose of this analysis, these children were still classified as non‐responders. While a maximum of 16 weeks of treatment is standard in many current CMAM protocols, we allowed an extra week because some programmes provide treatment every 2 weeks and children may have missed attendance at one or more follow‐up visits. Children who did not reach the recovery criteria set here but who were discharged as recovered based on their programme definition (often either WHZ ≥−2 or MUAC ≥12.5, rather than both) are classified as “early discharge” for the purpose of this analysis.

Due to the varying follow‐up frequencies across datasets, follow‐up intervals were set according to a 7‐day interval for weekly visits (severely wasted cases were applied to this schedule if not specified) or a 14‐day interval for visits every 2 weeks (moderately wasted cases were applied to this schedule if not specified).

Z‐score outliers were cleaned according to World Health Organisations' (WHO) recommended cleaning method for survey data (see definition in Appendix Table S1) (WHO, 2009). As this method is likely to exclude some legitimately extremely malnourished children, we also ran the analysis with cleaning based on a boxplot method which used the internal interquartile range of the data (Appendix Table S1) (Hoaglin et al., 1986). Children with nutritional oedema were included and analysed separately for most analyses.

### 2.3 Data analysis

Analyses were conducted in Stata v14.2 (StataCorp). Simple proportions and means were calculated to describe the data and assess the prevalence of WAZ <−3 in the combined sample, and within different admission types (severe wasting without oedema, severe wasting with oedema, moderate wasting [no moderate wasting with oedema as by definition oedema was classified as severe]). A calculation of percentage increase in caseload if children with WAZ <−3 were all treated within an outpatient therapeutic programme was calculated.

For comparing binary treatment outcomes between children admitted with and without WAZ <−3, logistic regression was used to generate odds ratios (OR). For continuous treatment outcomes (length of stay and average weight gain), linear regression was used among those who recovered. For all regression analyses, adjustment for the cluster by dataset was applied and a *p* value less than 0.05 was considered statistically significant.

We sought to explore whether the intensity of treatment received (based on supplementary food composition and dosage) affected treatment outcomes for children with WAZ <−3, recognising that with multiple different treatment protocols in a pooled analysis, the food composition and dosage varied across included datasets. We considered the receipt of home foods or fortified blended flours to be “low‐intensity treatment”, and receipt of lipid nutrient supplements (LNS) dosed at approximately 75 kcal/kg of body weight per day or approximately 500 kcal per day to be “mid‐intensity treatment”, and receipt of LNS dosed at approximately 175 kcal/kg of body weight per day or approximately 1000 kcal per day to be “high‐intensity treatment”. LNS includes ready‐to‐use therapeutic food (RUTF) and ready‐to‐use supplementary food (RUSF), both standard formulations currently used in wasting treatment programming and experimental formulations. We used logistic regression to compare treatment outcomes for those who received low‐intensity treatment versus mid‐intensity treatment, and low versus high‐intensity treatment, disaggregated by WAZ admission status (see further definition in Appendix Table S2). The model was adjusted for clustering by dataset and potential confounding factors: age, sex, and wasting severity at admission.

As there is currently no gold standard definition of recovery from wasting, and the definition applied here is still only a proxy for “health”, a large part of our analysis focuses on visual and statistical comparisons of growth trajectories to examine the response to treatment for different groups. Timepoints with measurements from fewer than 50 children were not included in plots, hence most trajectories track mean anthropometry and 95% confidence intervals from admission, every 7 or 14 days, until 126 days. Plots compare children with WAZ <−3 and children with WAZ ≥−3 for both the whole sample, as well as disaggregated by our definitions of severe wasting, moderate wasting, and nutritional oedema.

## 3 RESULTS

Datasets from 13 studies were included, collected between 2010 and 2020, covering 9 countries across Africa and Asia (Appendix Table S3). The original study methods are fully described in the original publications (Bahwere, Akoma et al., 2017; P. Bahwere, Balaluka et al., 2016; Bailey et al., 2020; Bhandari et al., 2016; Binns et al., 2016; Chase et al., 2020; Hsieh et al., 2015; Karakochuk et al., 2012; LaGrone et al., 2012; Oakley et al., 2010; Sigh et al., 2018; Stobaugh et al., 2016; Trehan et al., 2013).

Following the cleaning of extreme outliers, 783 (2.9%) children with missing WAZ at admission and 965 (3.6%) children who were not considered wasted based on our definitions and did not have oedema were excluded. Some children (21%) who we classified as severely wasted were treated in a supplementary feeding programme and some children (38%) who we classified as moderately wasted were treated in a therapeutic feeding programme (Appendix Table S4). A large proportion of these was due to different admission criteria for supplementary feeding in the Karakochuk et al.s' (2012) study.

### 3.1 Baseline demographics

The final pooled datasets contained data from 24,829 children aged 6–59 months. Females made up 53.5% of the sample (Table 1). The median age of the study participants was 18 months. At admission, 38.9% of the children had severe wasting without oedema and 42.6% of the children had moderate wasting. Approximately 14% of the children admitted for treatment had severe wasting defined by oedema only, while 4.2% had both oedema and WHZ <−3 or MUAC <11.5 cm. The largest proportion (43.9%) of the children in the sample resided in Malawi. Of those with oedema (4411), 96% were in Malawi with very few in other locations. Of the interventions received, 30.8% of children received standard formula RUTF while 4.3% of the children received standard RUSF (Table 1). Approximately 78.0% of children had one or more reported morbidities (fever, ARI, diarrhoea, vomiting, rash) during the treatment period. Regarding the intensity of treatment, 55.8% of the sample received therapeutic treatment with LNS (high intensity), 23.9% received a supplementary dose of LNS (mid‐intensity), and 20.3% received fortified flour or energy‐dense home foods (low‐intensity treatment).

**Table 1:** Characteristics of children treated for wasting (*n* = 24,829)

Attribute	Frequency (*n*)	Percent (%)
Sex		
Male	11,764	47.4
Female	13,065	52.6
Age (months), median (IQR)	18 (11, 28)	
Age groups (months)		
6–23	16,310	65.69
24–59	8519	34.31
Admission status		
Severely wasted		
by low MUAC and/or WHZ	9660	38.91
by oedema only	3537	14.25
by both oedema and low MUAC and/or WHZ	1048	4.22
Moderately wasted	10,584	42.63
HIV status at admission		
Unknown	21,913	88.26
Negative test	2771	11.16
Positive test^a^	145	0.58
Morbidity (diarrhoea, cough, fever, vomiting and/or rash) before or during treatment		
No	3472	21.72
Yes	12,492	78.16
Unknown	18	0.11
Type of research		
RCT (experimental)	18,623	75.01
Observational (routine)	6206	24.99
Country		
Malawi	10,902	43.91
Cambodia	113	0.46
Ethiopia	2572	10.36
DR Congo	406	1.64
India	836	3.37
Yemen	815	3.28
South Sudan	4355	17.54
Chad	1696	6.83
Kenya	3134	12.62
Type of intervention received		
Standard RUTF	10,367	41.75
Novel RUTF formulation	4687	18.88
CSB/CSB++	1985	7.99
Standard RUSF	1070	4.31
Novel RUSF formulation	4207	16.94
Home foods	277	1.12
Unknown supplementary food	2236	9.01

Abbreviations: CSB, cord‐soy blended flour; DR Congo, Democratic Republic of Congo; RCT, randomised controlled trial; RUSF, ready‐to‐use supplementary food; RUTF, ready‐to‐use therapeutic food.

^a^ HIV testing was uncommon and could reflect maternal status in those younger than 18 months.

Among this sample, severe underweight (WAZ <−3) was the most prevalent anthropometric deficit (55.0%) followed by severe stunting (HAZ <−3) (38.8%) (Appendix Table S5). The prevalence of WaSt (WHZ <−2 and HAZ <−2) was 43.5%; 48.2% among children with severe wasting and 37.2% among children with moderate wasting.

### 3.2 Describing the severely low WAZ (<−3) admission group

The overall prevalence of WAZ <−3 was 55.0% in children admitted for treatment (Table 2). Of moderately and severely wasted admissions, 42.8% and 64.0% had WAZ <−3, respectively. WAZ <−3 was more frequent in males (63.7%) than in females (47.1%), and more frequent in older children (66.3% aged 24–59 months) than in younger children. Among children with oedema, 30.7% had WAZ <−3 and 83.9% of children with both wasting and oedema had WAZ <−3. The prevalence of WAZ <−3 varied by location, ranging between 32.1% in Kenya and 89.7% in India.

**Table 2:** Prevalence of WAZ <−3 among children admitted to CMAM

Attribute	Total (*n*)	WAZ <−3 all admissions (%)	WAZ <−3 among MW (%)	WAZ <−3 among SW^a^ (%)
			*n* = 10,584	*n* = 14,245
Overall	24,829	54.96	42.78	64.00
Sex				
Male	11,764	63.72	52.07	70.40
Female	13,065	47.06	36.45	56.93
Age				
6–23	16,310	49.06	35.34	60.27
24–59	8519	66.25	59.59	70.36
Morbidity				
HIV‐positive	145	59.30	55.60	59.60
Morbidity before treatment	12,492	55.73	45.39	62.46
Country				
Malawi	10,902	54.27	48.91	57.40
Cambodia	113	62.83	31.91	85.85
Ethiopia	2572	60.93	48.13	70.89
DR Congo	406	67.49	100	67.25
India	836	89.71	79.46	91.30
Yemen	815	44.54	41.03	53.48
South Sudan	4355	60.34	39.00	68.80
Chad	1696	63.09	58.76	75.00
Kenya	3134	32.10	20.73	59.10

Abbreviations: CMAM, community‐based management of acute malnutrition; MW, moderate wasting; WAZ, weight‐for‐age z‐score.

a Severely wasted (SW) cases include those with oedema.

According to this data, the caseload of children warranting treatment with a therapeutic dose (as for severe wasting or oedema) would increase by 31.8% if those with both moderate wasting and WAZ <−3 were added to therapeutic treatment programmes (Appendix Table S6). Note that the increase in caseload calculated from this data represents the minimum increase, resulting only from shifting children from supplementary feeding to therapeutic feeding treatment. This estimate also relies on the proportions of moderate and severely wasted children recruited into the programmes reflecting the proportions in the community.

### 3.3 Comparing those admitted with and without severely low WAZ (<−3)

Recovery was lower in children with WAZ <−3 compared to children with WAZ ≥−3 (28.3% vs. 48.3%). The proportion of children who died was also higher in severely low WAZ children (1.8% vs. 0.8%), but non‐response and defaulter rates were similar between the two groups (Appendix Table S7). Approximately 64% of children with WAZ <−3 were also severely stunted (HAZ <−3) compared to 10% of children with WAZ ≥−3.

When disaggregated by severe wasting, moderate wasting, or oedema, severely underweight children had lower odds of recovery than children with WAZ ≥−3 across all three of these groups (Table 3). Children admitted with oedema at WAZ <−3 demonstrated the largest reduction in recovery compared to those with oedema and WAZ ≥−3 (OR = 0.24). For severely wasted children, those with WAZ <−3 had significantly greater odds of death than those without low WAZ. The highest death rate was in the sub‐group of oedematous children with severely low WAZ (5.7%). Across all admission groups, the odds of being transferred to alternative treatment, including inpatient care, were significantly greater among severely underweight children. When all adverse outcomes were combined (deaths, defaulters, non‐response, and transfer), there were significantly greater odds of adverse outcomes for those with WAZ <−3 compared to those with WAZ ≥−3 for children with moderate wasting or oedema (Table 3). Among children who did recover, those who began with severely low WAZ had similar lengths of stay as children with WAZ ≥−3, except for those with oedema. Results were similar when using the “boxplot” data cleaning method which excludes fewer children with extreme Z‐scores (Appendix Table S8). The child's age had a significant positive effect on recovery but this did not differ by WAZ <−3 status.

**Table 3:** Comparison of treatment outcomes for those with and without WAZ <−3, disaggregated by admission status

	SW without oedema (*n* = 9660)				SW with oedema (*n* = 4585)				MW (*n* = 10,584)			
Outcome *n* (%)	WAZ <−3 *n* (%) (*n* = 7153)	WAZ ≥−3 *n* (%) (*n* = 2507)	Odds ratio^a^ (95% CI)	*p* value	WAZ <−3 *n* (%) (*n* = 1964)	WAZ ≥−3 *n* (%) (*n* = 2621)	Odds ratio^a^ (95% CI)	*p* value	WAZ <−3 *n* (%) (*n* = 4528)	WAZ ≥−3 *n* (%) (*n* = 6056)	Odds ratio^a^ (95% CI)	*p* value
Recovered^b^	1274 (17.81)	503 (20.06)	0.70 (0.62, 0.80)	<0.001	954 (48.57)	2075 (79.17)	0.24 (0.21, 0.28)	<0.001	1630 (36.00)	2820 (46.57)	0.59 (0.54, 0.64)	<0.001
Died	107 (1.50)	9 (0.36)	2.56 (1.27, 5.17)	0.008	111 (5.65)	52 (1.98)	2.95 (2.11, 4.12)	<0.001	29 (0.64)	25 (0.41)	1.73 (0.99, 3.03)	0.055
Defaulted	886 (12.39)	364 (14.52)	1.09 (0.94, 1.26)	0.243	95 (4.84)	80 (3.05)	1.74 (1.27, 2.38)	0.001	307 (6.78)	644 (10.63)	1.08 (0.92, 1.27)	0.339
Non‐response	1755 (24.54)	884 (35.26)	0.84 (0.75, 0.94)	0.004	120 (6.11)	65 (2.48)	2.71 (1.92, 3.84)	<0.001	929 (20.52)	1202 (19.85)	1.08 (0.96, 1.23)	0.208
Transfer	434 (6.07)	95 (3.79)	1.55 (1.23, 1.96)	<0.001	69 (3.51)	45 (1.72)	1.94 (1.32, 2.85)	0.001	342 (7.55)	284 (4.69)	1.24 (1.05, 1.47)	0.011
Early discharge^c^	2169 (30.32)	568 (22.66)	1.25 (1.11, 1.42)	<0.001	608 (30.96)	303 (11.56)	3.54 (3.02, 4.14)	<0.001	1186 (26.19)	956 (15.79)	1.66 (1.49, 1.84)	<0.001
Unknown outcome^d^	528 (7.38)	84 (3.35)	1.25 (0.94, 1.66)	0.111	7 (0.36)	1 (0.04)	12.65 (1.35, 118.7)	0.026	112 (2.47)	131 (2.16)	1.11 (0.83, 1.47)	0.486
All adverse outcomes^e^	3182 (44.48)	1352 (53.93)	1.03 (0.93, 1.15)	0.570	1600 (35.34)	2149 (35.49)	1.22 (1.09, 1.35)	<0.001	395 (20.11)	242 (9.23)	2.54 (2.12, 3.05)	<0.001

**Response** f	**Mean (±*SD*)**	**Mean (±*SD*)**	**Mean difference** g **(95% CI)**	***p* value**	**Mean (±*SD*)**	**Mean (±*SD*)**	**Mean difference** g **(95% CI)**	***p* value**	**Mean (±*SD*)**	**Mean (±*SD*)**	**Mean difference** g **(95% CI)**	***p* value**
Length of stay (days)	65.11 ± 34.04	61.93 ± 32.27	3.17 (−9.59, 15.93)	0.606	32.74 ± 17.11	27.26 ± 15.26	5.48 (2.67, 8.30)	0.002	44.70 ± 30.71	44.90 ± 28.89	−0.21 (−6.09, 5.67)	0.941
Weight gain (g/kg/day)	4.14 ± 5.43	3.26 ± 3.48	0.87 (−0.29, 2.04)	0.132	4.26 ± 2.52	3.10 ± 2.11	1.16 (1.02, 1.30)	<0.001	3.55 ± 4.08	2.67 ± 2.95	0.89 (0.23, 1.54)	0.011

Abbreviations: CI, confidence interval; MUAC, mid‐upper‐arm circumference; MW, moderately wasted; SW, severely wasted; WAZ, weight‐for‐age z‐score.

^a^ Odds ratio is bivariate association between WAZ <−3 with reference to WAZ ≥−3.

^b^ Children who had attained nutritional recovery at discharge (MUAC ≥12.5 and WHZ ≥−2 and no oedema) within a period of 17 weeks of admission. These recovery criteria were not necessarily the same as those used in the individual programmes and therefore should not be used as indicators of programme quality.

^c^ Children who were discharged as cured but had not attained nutritional recovery, as per our definition.

^d^ Children with missing treatment outcomes.

^e^ All adverse outcomes combines deaths, defaulters, non‐response, and transfer.

^f^ Length of stay and weight gain among recovered children only.

^g^ Linear regression analysis.

### 3.4 Growth curve analysis

**Figure 1:**
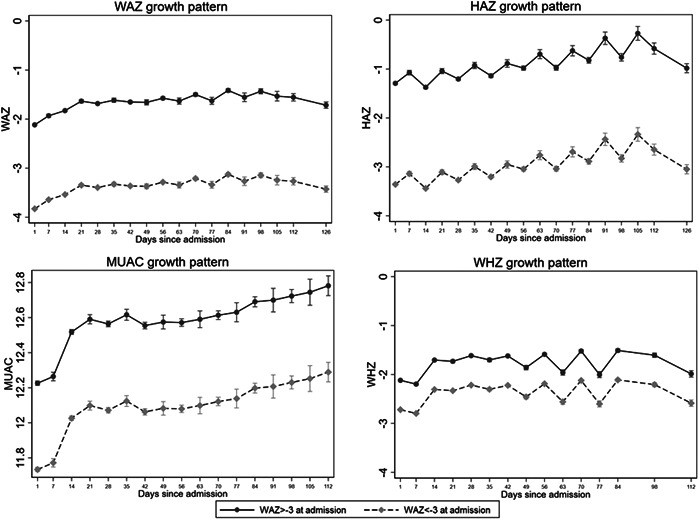
Growth trajectories for WAZ, HAZ, MUAC and WHZ, disaggregated by WAZ status at admission. MUAC, mid‐upper‐arm circumference; WAZ, weight‐for‐age z‐score

While there was a clear difference in the starting anthropometry of those admitted with WAZ <−3 and those with WAZ ≥−3, the growth trajectories (changes in WAZ, HAZ, MUAC and WHZ) during treatment were all very similar (Figure 1). The low WAZ group started with greater deficits across all growth indicators and remained with almost identical growth deficits at each timepoint and at the end of treatment. In general, severely wasted admissions tended to have steeper gains in all anthropometric indicators than children with moderate wasting (graphs disaggregated by wasting severity and oedema status in Appendix Figures S1–S4). The children with moderate wasting had an especially flat growth curve when considering HAZ. The greatest gains in HAZ were seen in children with oedema; this group generally had the greatest HAZ deficits at admission, had a less severe deficit in MUAC and WHZ at admission, and received high‐intensity treatment. Overall, change in HAZ was significantly higher for low WAZ admissions (0.36 vs. 0.17) (mean difference: 0.19, 95%  confidence interval: 0.16–0.22, *p* < 0.0001). Older children were significantly less likely to have gains in HAZ but this did not differ by WAZ <−3 status.

### 3.5 Intensity of nutrition treatment

**Figure 2:**
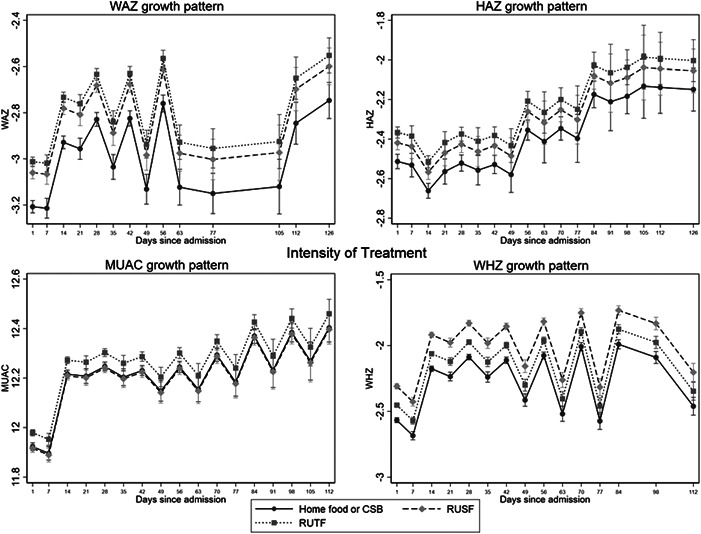
Growth trajectories for different intensities of treatment Low‐intensity treatment = home foods/corn/soy blended flour (CSB). Mid‐intensity treatment = supplementary food (RUSF), high intensity treatment = therapeutic food (RUTF). Note, these curves are not adjusted for severity of deficit at admission, whereas the logistic regression in of treatment outcomes Appendix Table S9 is.

After adjusting for age, sex and severity of wasting at admission, mid‐intensity treatment had a superior recovery rate compared to low‐intensity treatment (Appendix Table S9). High‐intensity treatment (therapeutic dose of RUTF) had a significantly lower defaulter rate than low‐intensity treatment, but greater odds of non‐response. The growth trajectories (Figure 2) show very similar growth patterns across the different intensities of treatment. Those with WAZ ≥−3 may respond better to current options of mid‐ and high‐intensity treatment than those with WAZ <−3 (recovery rate for low‐mid‐high intensity for WAZ <−3: 17%, 39%, 25%; for WAZ ≥−3: 24%, 61%, 43%).

## 4 DISCUSSION

The purpose of this study was to draw on existing research and programmatic data to better understand growth trajectories and response to treatment of children with WAZ <−3 within wasting treatment programmes. A significant proportion of wasted children had severely low WAZ (approximately 40% of moderately wasted admissions and 60% of severely wasted admission), however, these proportions varied greatly by context. Children admitted with WAZ <−3 had lower recovery rates, higher risk of death, and higher risk of transfer to inpatient care, both overall and when disaggregated by oedema/severity of wasting. Children admitted with severely low WAZ had markedly lower anthropometric indicators (HAZ, WHZ and MUAC) at both admission and end of care; however, their patterns of anthropometric gains were very similar to those with WAZ ≥−3. Among children that fully recovered, the length of stay was the same for WAZ <−3 and WAZ ≥−3 admissions, but the rate of weight gain was slightly faster for the lower WAZ group.

These results suggest that firstly wasted children with WAZ <−3 do have additional vulnerabilities and greater anthropometric deficits all around which likely impacts their risk of death during treatment indicating that they should indeed be targeted for increased support. We found that there are some very high‐risk children within the severely low WAZ group, as evidenced by the higher death rate despite being enrolled in CMAM support. This is especially relevant for children with moderate wasting and WAZ <−3 (of which 0.64% died), as these children would not be eligible for support in many settings where there is no moderate wasting treatment programme. In such settings or where resources are limited, children with moderate wasting and WAZ <−3 should be prioritised for nutritional support, alongside those with severe wasting. A study of children with moderate wasting provided with nutrition counselling in Sierra Leone found WAZ <−3 to be a significant predictor of death and deterioration to severe wasting (Lelijveld et al., 2021). In settings where WAZ is routinely assessed, connections with wasting treatment programmes should be enhanced.

We also found that children with oedema and severely low WAZ were the highest risk group in this analysis with regard to survival; they were also the most stunted group at admission. Evidence already highlights that children who are both wasted and oedematous (“marasmic‐kwashiorkor”) are at high risk of mortality (Scrimshaw & Viteri, 2010). However, this has not traditionally included considerations of stunting, nor is there any increase in support in standard treatment programming for these children. This group warrants particular attention and further work is needed to uncover the cause of their vulnerability to mitigate it.

Secondly, while wasted children with WAZ <−3 have poorer recovery rates, they are responding well to CMAM treatment, despite starting treatment with greater deficits. However, their anthropometry remains well below the WAZ ≥−3 group at discharge, even after several months in the highest intensity treatment (RUTF). These findings, therefore, pose important questions about what the appropriate definitions of “recovery” should be, especially in these children with very low and multiple anthropometric deficits at admission, and what the most appropriate treatment should be. Longer or higher intensity treatment may be necessary for a more complete and sustained recovery in children with the greatest deficits, and the commonly used definition of “non‐response” (after 16 weeks of treatment) may need to be extended. It is also important to note that our data lacks contextual information on co‐morbidities, access to care, and family status which may influence a child's response to food treatment and should be considered in programming.

In general, the recovery rates, based on our stringent definition were low. Discharge criteria vary considerably across treatment protocols and this has a large impact on reported recovery rates (Guesdon & Roberfroid, 2019). It is important to note that binary anthropometric thresholds do not equate to nutritional recovery in all children or in all contexts. Rather, anthropometry is a sliding scale proxy for near‐term mortality risk, and there are a variety of other recovery definitions that could be applied. We also know that anthropometric recovery after wasting does not equate to immune recovery and that postdischarge relapse, morbidity, and mortality remain high, even in those discharged as fully recovered (Kerac et al., 2014; Njunge et al., 2020; Stobaugh et al., 2019). Given that we see anthropometric gains in children with WAZ <−3 but lower recovery rates based on MUAC and WHZ, it may be appropriate to define recovery based on WAZ for these admissions. A recent secondary analysis found similarly worse treatment outcomes in children admitted with severely low WAZ (<−3) when data from the Combined Protocol for Acute Malnutrition Study (ComPAS) cluster randomised controlled trial in Kenya and South Sudan (Bailey et al., 2020, 2021) were analysed.

Lastly, our analysis found that both children with WAZ <−3 and with WAZ ≥−3 can have modest gains in HAZ during CMAM treatment. This is a more positive picture than seen in a follow‐up of children who recovered from severe wasting in Niger, who saw slight reductions in HAZ both during and after treatment (Isanaka et al., 2019). We found that those with the greatest deficits in HAZ had the greatest gains, as has been reported previously (Rao & Naidu, 1977). The flattest slope in HAZ growth plots was for children admitted with moderate wasting, and these children are more likely to receive a lower intensity of treatment. There is evidence that the high bioavailability of essential amino acids is necessary to activate signalling pathways and growth hormones necessary for catch‐up growth (Semba et al., 2016). A study of reduced dosage of therapeutic food for children with wasting in Burkina Faso saw weight gain similar to standard dosage, but reduced linear growth velocity (Kangas et al., 2019). This suggests a minimum intensity or quality of treatment may be required to promote linear growth, or at least maintain HAZ, in children with wasting, especially in those with severely low WAZ.

### 4.1 Limitations

This analysis does not capture all children with severely low WAZ, only those with wasting and severely low WAZ. Those children with severely low WAZ who are not wasted must therefore be severely stunted and are likely to be at particularly high risk of death (Myatt et al., 2019). Prospective data on the response to treatment of children with WAZ <−3 but not captured by current wasting programmes are urgently needed. This limitation also affects the calculated increases in caseload which are, therefore, an underrepresentation. Caseload calculations also assume that the proportion of severely and moderately wasted children in the datasets represents the natural proportions of caseloads presenting for treatment, which is not necessarily true given the likely differences in coverage and recruitment practices. This analysis is also limited in its geographical generalisability given the low representation in data from Asia, Latin America and Oceania, and it only focuses on children over 6 months of age despite infants under 6 months also having a high prevalence of underweight and poor outcomes. Lastly, the results of treatment outcomes are affected by variations in protocols between the studies, particularly treatment protocols and admission/discharge criteria. There is also heterogeneity within the intensity of treatment categories, such as experimental vs standard LNS. Applying a standard anthropometric definition of recovery across the datasets, which is often more stringent than those used in the original studies, results in a large proportion (between 12% and 30%) of children being defined as “early discharge”. We do not know what the outcome for these children would have been, had the programmes continued to treat them based on the definition applied in this analysis. An alternative for overcoming this limitation is to analyse the data using the various outcome definitions that were applied by the original studies (discharge criteria for each study are presented in Appendix Table S3). When this analysis is run (Appendix Table S10 and S11), our conclusions remain the same, however, the effect size of poor outcomes for those with WAZ < ‐3 is diminished, which may be important to consider for any future sample size calculations.

## 5 CONCLUSION

This analysis supports evidence that children with wasting and WAZ <−3 are even more vulnerable to mortality and do not achieve traditional definitions of nutritional recovery as often as other wasted children. Wasted children with WAZ <−3 do gain weight in a similar pattern to other children with wasting, however, they have further to catch up. Further field studies are needed to explore appropriate definitions of nutritional recovery, non‐response cut‐offs, the appropriate intensity of treatment, and the appropriate length of support required for treating children with multiple anthropometric deficits. This analysis also suggests that for wasted children with severely low WAZ, longer or higher intensity treatment may be needed. This is especially true for children with moderate wasting and WAZ <−3, who are only eligible for low‐intensity treatment in many settings. Children with severely low WAZ plus oedema likely require the greatest support due to the highest mortality risk. An intervention trial is needed to further explore these hypotheses and must include nonwasted children with WAZ <−3, who are currently not represented in available data from CMAM programmes.

## AUTHOR CONTRIBUTIONS

**Heather Stobaugh**: created the call for data and conceived the study. **Heather Stobaugh, Gloria A. Odei Obeng‐Amoako, Natasha Lelijveld, Stephanie V. Wrottesley and Tanya Khara**: detailed the protocol. **All authors**: reviewed the protocol and methods. **Gloria A. Odei Obeng‐Amoako**: conducted the data cleaning and merging. **Gloria A. Odei Obeng‐Amoako and Natasha Lelijveld**: conducted the data analysis. **Gloria A. Odei Obeng‐Amoako, Natasha Lelijveld and Stephanie V. Wrottesley**: wrote the first draft of the manuscript with regular contributions from all authors. **All authors**: read and approved the final manuscript.

## CONFLICTS OF INTEREST

The authors declare no conflicts of interest.

## Supporting information

Supporting information.

## Data Availability

The full dataset cannot be made open access due to the presence of participant identifiable content. However, underlying data, code and supporting documentation for this paper are available by request. A redacted version will be provided to interested parties, subject to the signing of Data Transfer Agreements with each of the original principal investigators. Inquires can be made to office@ennonline.net.
