# Supplementary material for: How do children with severe underweight and wasting respond to treatment? A pooled secondary data analysis to inform future intervention studies
Source: Matern Child Nutr. 2022 Oct 19;19(1):e13434. doi: 10.1111/mcn.13434 (PMC9749592; doi:10.1111/mcn.13434)
Supplement: Supplementary file 1 — Supporting information. [file MCN-19-e13434-s001.docx]

**Supporting Information for: How do children with severe underweight and wasting respond to treatment? A pooled secondary data analysis to inform future intervention studies**

**Appendix Table 1. Comparison of implausible Z-score censoring methods**

|  | **WHO method** | | **Boxplot (IQR) method^§^** | |
| --- | --- | --- | --- | --- |
|  | **Thresholds** | **Number of children excluded** | **Thresholds** | **Number of children excluded** |
| WAZ | <-6 & >+5 | 778 | < -9.14 & >2.83 | 172 |
| HAZ | <-6 & >+6 | 2,222 | < -11.20 & >5.93 | 584 |
| WHZ | <-5 & >+5 | 738 | <-7.20 & >2.25 | 363 |
| **^§^**±3* IQR (interquartile range) | | | | |

**Appendix Table 2: definition of low-, mid- and high-intensity treatment in this study**

| **Treatment category** | **Type of treatment provided** |
| --- | --- |
| **Low intensity treatment** | Home foods or fortified blended flours (CSB, SuperCereal plus) |
| **Mid intensity treatment** | Supplementary dose of lipid nutrient supplements (LNS e.g. RUSF)  (~75 kcal/kg/day or 1 sachet per day) |
| **High intensity treatment** | Therapeutic dose of lipid nutrient supplements (LNS e.g, RUTF)  (~175 kcal/kg/day or 2 sachets per day)* |

*For the purpose of this analysis, the LNS dosages provided by in simplified protocol trials are considered ‘high-intensity’ as they provided a therapeutic dose for children with severe wasting and/or oedema. RUSF=ready-to-use supplementary food; RUTF= ready-to-use therapeutic food.

**Appendix Table 3: Details of studies that contributed data to this pooled analysis**

| **Original study reference** | **Country** | **Study design** | **Year of data collection** | **N** | **Programme type** | **Admission criteria** | **Discharge criteria** | **Intervention** |
| --- | --- | --- | --- | --- | --- | --- | --- | --- |
| Bahwere et al., 2016 | Democratic Republic of Congo | Randomized, controlled trial | 2013-2014 | 886 | Therapeutic | MUAC <11.5 cm or oedema | MUAC ≥12.5 cm and no oedema for 15 consecutive days | Soya, maize, and sorghum RUTF vs. standard RUTF |
| Bahwere et al., 2017 | Malawi | Randomized, controlled trial | 2015-2016 | 1347 | Therapeutic | MUAC <11.5 cm or oedema | MUAC ≥12.5 cm and no oedema | Milk-free, soya, maize, and sorghum RUTF vs. milk, soya, maize and sorghum RUTF |
| Bailey et al., 2020 | South Sudan, Kenya | Cluster randomized, controlled trial | 2017-2018 | 2488 | Both therapeutic and supplementary | MUAC <12.5 cm or oedema | MUAC ≥12.5 cm and no oedema for 2 consecutive visits | Combined, simplified protocol (RUTF) vs. standard protocol (weight-based RUTF for SW or RUSF for MW) |
| Bhandari et al., 2016 | India | Randomized, controlled trial | 2012-2015 | 906 | Therapeutic (but includes home foods) | WHZ <−3 or oedema | WHZ ≥−2 and no oedema | Standard RUTF vs. locally prepared RUTF vs. energy-dense home foods |
| Binns et al., 2014 | Malawi | Standards-based trial | 2011-2012 | 258 | Therapeutic | MUAC <11.5 cm and no oedema | MUAC ≥12.5 cm and no oedema for two consecutive visits | Standard treatment as per national guidelines (RUTF and amoxicillin) |
| Chase et al., 2020 | Yemen, South Sudan, Kenya, Chad | Retrospective analysis of routine program data | 2010-2016 | 8233 | Both therapeutic and supplementary | WHZ <−2 and/or MUAC <12.5 cm or oedema | As per national protocols – varied by country | As per national protocols: 200 kcal/kg/day RUTF for SW and supplementary foods for MW |
| Hsieh et al., 2015 | Malawi | Prospective, randomized, double-blinded, clinical trial | 2014 | 141 | Therapeutic | MUAC <11.5 cm and/or oedema | MUAC >12.4 cm and no oedema | High oleic acid RUTF vs. standard RUTF (~175 kcal/kg/day) for up to 12 weeks |
| Karakochuk et al., 2012 | Ethiopia | Cluster-randomized trial | 2009 | 1125 | Supplementary | MUAC <13.5 cm and WH ≥70 and <80% (NCHS) | WH ≥85% (NCHS) on two consecutive visits | RUSF vs. CSB for up to 16 weeks |
| LaGrone et al., 2012 | Malawi | Prospective, randomized, controlled noninferiority trial | 2009-2010 | 2712 | Supplementary | WHZ <-2 and ≥-3 without oedema | WHZ ≥-2 | CSB++ vs. locally produced soy RUSF vs. imported soy/whey RUSF (~75 kcal/kg/day) for up to 12 weeks |
| Oakley et al., 2010 | Malawi | Randomized, double-blind, clinical, quasi-effectiveness trial | 2008-2009 | 1874 | Therapeutic | WHZ <-3 and/or edema | WHZ ≥-2 and no oedema | 25% milk RUTF vs. 10% milk RUTF (~175 kcal/kg/day) for up to 8 weeks |
| Stobaugh et al., 2016 | Malawi | Prospective, randomized, double-blinded, controlled clinical trial | 2013-2014 | 2230 | Supplementary | MUAC ≥11.5 & <12.5 cm without oedema | MUAC ≥12.5 cm for two consecutive visits and no oedema | Soy RUSF vs. whey RUSF (~75 kcal/kg/day) for up to 12 weeks |
| Sigh et al., 2018 | Cambodia | Prospective, randomized, home-based trial | 2015-2017 | 121 | Therapeutic | WHZ ≤-2.8 or MUAC ≤11.5 cm and/or oedema | WHZ ≥-2 and MUAC >11.5 cm and no oedema | Locally produced fish-based RUTF vs. standard RUTF for up to 8 weeks |
| Trehan et al., 2013 | Malawi | Prospective, randomized, double-blind, placebo-controlled clinical trial | 2009-2011 | 2767 | Therapeutic | WHZ <-3 and/or oedema | WHZ ≥-2 without oedema | RUTF (~175 kcal/kg/day) for up to 12 weeks and amoxicillin or cefdinir vs. RUTF and placebo for 7 days |

Abbreviations: CSB, corn-soya blend; MUAC, mid-upper arm circumference; RUSF, ready-to-use supplementary food; RUTF, ready-to-use therapeutic food; WHZ, weight-for-height z-score. WH, weight for height. Oedema= bilateral pitting oedema. SW, severe wasting; MW, moderate wasting

**Appendix Table 4: Anthropometric classifications vs intensity of treatment received**

|  | **Therapeutic feeding*** | **Supplementary feeding*** |
| --- | --- | --- |
| SAM by MUAC or WHZ | 6703 | 2957 |
| MAM | 4052 | 6532 |
| SAM with oedema | 4574 | 9 |
| No wasting | 380 | 585 |

*therapeutic feeding= ready-to-use therapeutic food at 175kcal/kg/day; supplementary feeding= lipid nutrient supplement at a dose of 75kcal/kg/day or home foods or fortified blended flours. SAM= severe acute malnutrition defined as mid-upper arm circumference (MUAC)<11.5cm or weight-for-height z-score (WHZ) <-3. MAM= moderate acute malnutrition defined as MUAC<12.5 and ≥11.5cm or weight-for-height z-score (WHZ) <-2 and ≥-3.

**Appendix Table 5: Nutritional status/anthropometric deficits at admission**

| **Nutritional status* n (%)** | **WAZ (n=24,829)** | **WHZ (n=24,196)** | **HAZ (n=23,115)** | **MUAC (n=24,755)** |
| --- | --- | --- | --- | --- |
| **Normal**  (z-scores ≥-2; MUAC ≥12.5cm) | 4,467 (17.99) | 7,179 (29.67) | 8,325 (36.02) | 4,905 (19.81) |
| **Moderately low**  (z-scores -2 to -3; MUAC 11.5 to 12.5cm) | 6,717 (27.05) | 9,842 (40.68) | 5,813 (25.15) | 13,232 (53.45) |
| **Severely low**  (z-scores <-3; MUAC<11.5cm) | 13,645 (54.96) | 7,175 (29.65) | 8,977 (38.84) | 6,618 (26.73) |

*WAZ: weight-for-age z-score, WHZ: weight-for-height z-score, HAZ: height-for-age z-score, MUAC: mid-upper arm circumference

**Appendix Table 6 Percentage increase in severe wasting case load if WAZ<-3 was added as an additional inclusion criterion**

| **Attribute** | **Number of moderately wasted cases (n)** | **Number of severely wasted cases (n)** | **Moderate cases with low WAZ at admission (n, %)** | **% increase in admissions if MAM with WAZ<-3 added as a SAM treatment criterion*** |
| --- | --- | --- | --- | --- |
| **Overall** | 10,584 | 14,245 | 4,528 (42.78) | 31.79 |
| Male | 4,290 | 7,474 | 2, 234 (52.07) | 29.89 |
| Female | 6,294 | 6,771 | 2,294 (36.45) | 33.88 |
| 6-23 | 7,335 | 8,975 | 2,592 (35.34) | 28.88 |
| 24-59 | 3,249 | 5,270 | 1,936 (59.59) | 36.74 |
| Malawi | 4,026 | 6,876 | 1,969 (48.91) | 28.64 |
| Cambodia | 47 | 66 | 15 (31.91) | 22.73 |
| Ethiopia | 1,126 | 1,446 | 542 (48.13) | 37.48 |
| DR Congo | 3 | 403 | 3 (100) | 0.74 |
| India | 112 | 724 | 86 (79.46) | 11.88 |
| Yemen | 585 | 230 | 240 (41.03) | 104.35 |
| South Sudan | 1,236 | 3,119 | 482 (39.00) | 15.45 |
| Chad | 1,244 | 452 | 731 (58.76) | 161.73 |
| Kenya | 2,205 | 929 | 457 (20.73) | 49.19 |

*Note that this calculation is based on the proportions of SAM and MAM children being admitted to each particular programme and may not reflect standard or community ratios in case types.

**Appendix Table 7: Treatment outcomes by WAZ status**

| **Treatment Outcome** | **WAZ <-3 (n=13,645)** | **WAZ**≥ **-3 (n=11,184)** |
| --- | --- | --- |
|  | **n (%)** | **n (%)** |
| Defaulted | 1,288 (9.44) | 1,088 (9.73) |
| Died | 247 (1.81) | 86 (0.77) |
| Non responders* | 2,797 (20.50) | 2,145 (19.18) |
| Recovered** | 3,858 (28.27) | 5,398 (48.27) |
| Transfer | 845 (6.19) | 424 (3.79) |
| Unknown outcome | 647 (4.74) | 216 (1.93) |
| Early discharge*** | 3,963 (29.04) | 1,827 (16.24) |

*Non-responders included who had not attained nutritional recovery after 17 weeks in treatment, even though some of these children achieved recovery after 17 weeks.

**Only includes children who had attained nutritional recovery at discharge (based on both MUAC and WHZ and oedema status) within a period of 17weeks of admission

***Children who were discharged as cured but had not attained nutritional recovery based on definitions used in this study.

**Appendix Table 8:** **Treatment outcomes by WAZ status using ‘boxplot’ data cleaning method**

| **Treatment Outcome** | **Low WAZ (n=14,390)** | **Non-low WAZ (n=11,112)** | **Odds ratio** | **p- value** |
| --- | --- | --- | --- | --- |
|  | **n (%)** | **n (%)** |  |  |
| Defaulted | 1,412 (9.81) | 1,077 (9.69) | 1.36 | <0.001 |
| Died | 252 (1.75) | 86 (0.77) | 2.75 | <0.001 |
| Non responders* | 3,400 (23.63) | 2,589 (23.30) | 0.97 | 0.470 |
| Recovered** | 3,690 (25.64) | 4,928 (44.35) | 0.40 | <0.001 |
| Transfer | 903 (6.28) | 421 (3.79) | 1.60 | <0.001 |
| Unknown outcome | 730 (5.07) | 217 (1.95) | - | - |
| Early discharge*** | 4,003 (27.82) | 1,794 (16.14) | 2.11 | <0.001 |
| **Response** | **Mean (± SD)** | **Mean (± SD)** | **Coefficient** | **p-value** |
| Length of stay (days) | 50.56 ± 32.32 | 41.72 ± 28.09 | 8.83 | 0.051 |
| Weight gain (g/kg/day) | 3.94 ± 7.23 | 2.71 ± 2.53 | 1.23 | 0.005 |

N= 25,502 children based on this cleaning method. Prevalence of WAZ<-3 in this sample was 56.4%.

*Non-responders included who had not attained nutritional recovery after 17 weeks in treatment, even though some of these children achieved recovery after 17 weeks.

**Only includes children who had attained nutritional recovery at discharge (based on both MUAC and WHZ and oedema status) within a period of 17weeks of admission

***Children who were discharged as cured but had not attained nutritional recovery based on definitions used in this study

**Appendix Table 9: Treatment outcomes across difference intensities of treatment by admission weight**

|  | **Severely low WAZ (<-3)** | | | | | **WAZ**≥**-3** | | | | |
| --- | --- | --- | --- | --- | --- | --- | --- | --- | --- | --- |
|  | Low intensity  n  %  n=2,761 | Mid intensity  n  %  n=2,843 | High intensity  n  %  n=8,041 | Mid vs low  OR^  *P value* | High vs low  OR^  *P value* | Low intensity  n  % | Mid intensity  n  % | High intensity  n  % | Mid vs low  OR^  *P value* | High vs low  OR^  *P value* |
| Recovered | 479 (17.35) | 1,104  (38.83) | 2,275  (28.29) | **1.37**  ***0.001*** | 0.96  *0.739* | 421  (24.24) | 1,482  (60.89) | 3,039  (43.33) | **1.28**  ***0.013*** | 0.81  *0.20* |
| Dead | 14 (0.51) | 17 (0.60) | 216 (2.69) | 0.62  *0.259* | 1.00  *0.998* | 9 (0.52) | 5 (0.21) | 72 (1.03) | 0.40  *0.172* | 0.90  *0.868* |
| Defaulted | 219 (7.93) | 39 (1.37) | 1030 (12.81) | 0.65  *0.150* | **0.45**  ***0.001*** | 182 (10.48) | 49 (2.01) | 857 (12.22) | 0.54  *0.062* | **0.48**  ***<0.001*** |
| Non respondents | 783 (28.36) | 167 (5.87) | 1,850 (23.01) | 0.84  0.273 | **1.83**  **<0.001** | 605 (34.89) | 124 (5.09) | 1,893 (26.99) | **0.63**  ***0.007*** | **1.44**  ***0.002*** |
| Transfer | 201 (7.28) | 300 (10.55) | 344 (4.28) | 0.95  *0.736* | 1.03  *0.871* | 97 (5.58) | 156 (6.41) | 171 (2.44) | 1.28  *0.309* | **1.64**  ***0.026*** |
| Early discharge | 894 (32.38) | 1,207 (42.46) | 1,862 (23.16) | 0.89  *0.14* | 0.91  *0.53* | 382 (21.88) | 609 (24.86) | 1,292 (18.42) | 0.93  *0.48* | 0.90  *0.60* |

^Logistic regression accounting for clustering, adjusted for age, sex and SAM/MAM admission status. Bold indicates P<0.05. lowest intensity includes home foods and fortified blended flour; mid-intensity includes a supplementary dose of lipid nutrient supplements (RUTF or RUSF); high intensity includes a therapeutic dose of lipid nutrient supplement (RUTF).

**Appendix Table 10: Treatment outcomes (based on individual programme definitions) by WAZ status at admission**

| **Treatment Outcome** | **WAZ < -3 (n=** **13,645)** | **WAZ ≥-3 (n= 11,184)** | **Total (n=24,829)** |
| --- | --- | --- | --- |
|  | **n (%)** | **n (%)** | **n (%)** |
| Defaulted | 1,288 (9.44) | 1,088 (9.73) | 2,376 (9.57) |
| Died | 247 (1.81) | 86 (0.77) | 333 (1.34) |
| Non responders^*^ | 1,156 (8.47) | 722 (6.46) | 1,878 (7.56) |
| Recovered^*^ | 9,324 (68.33) | 8,490 (75.91) | 17,814 (71.75) |
| Transfer | 845 (6.19) | 424 (3.79) | 1,269 (5.11) |
| Unknown outcome^ǂ^ | 785 (5.75) | 374 (3.34) | 1,159 (4.67) |

*these outcomes include a number of definitions of recovery depending on the individual study protocols. ^ǂ^Children with missing treatment outcomes.

**Appendix Table 11: Comparison of treatment outcomes (based on individual programme definitions) for low WAZ vs non-low WAZ children, disaggregated by admission status**

|  | **SW without oedema (n=9,660)** | | | | **SW with oedema (n= 4,585)** | | | | **MW (n=10,584)** | | | |
| --- | --- | --- | --- | --- | --- | --- | --- | --- | --- | --- | --- | --- |
| **Outcome**  **n (%)** | **WAZ <-3**  **n (%)**  **(n=7,153)** | **WAZ ≥ -3**  **n (%)**  **(n=2,507)** | **Odds ratio^#^**  **(95% CI)** | **p- value** | **WAZ <-3**  **n (%)**  **(n=1,964)** | **WAZ** ≥ **-3**  **n (%)**  **(n=2,621)** | **Odds ratio^#^**  **(95% CI)** | **p value** | **WAZ <-3**  **n (%)**  **(n=4,528)** | **WAZ ≥ -3**  **n (%)**  **(n=6,056)** | **Odds ratio^#^**  **(95% CI)** | **p- value** |
| Recovered^*^ | 4,371 (61.11) | 1,619 (64.58) | 0.88 (0.79, 0.98) | 0.022 | 1,586 (80.75) | 2,403 (91.68) | 0.38 (0.32, 0.46) | <0.001 | 3,367 (74.36) | 4,468 (73.78) | 0.83 (0.75, 0.92) | <0.001 |
| Died | 107 (1.50) | 9 (0.36) | 2.56 (1.27, 5.17) | 0.008 | 111 (5.65) | 52 (1.98) | 2.95 (2.11, 4.12) | <0.001 | 29 (0.64) | 25 (0.41) | 1.73 (0.99, 3.03) | 0.055 |
| Defaulted | 886 (12.39) | 364 (14.52) | 1.09 (0.94, 1.26) | 0.243 | 95 (4.84) | 80 (3.05) | 1.74 (1.27, 2.38) | 0.001 | 307 (6.78) | 644 (10.64) | 1.08 (0.92, 1.27) | 0.339 |
| Non-response^*^ | 747 (10.44) | 288 (11.49) | 0.83 (0.71, 0.98) | 0.024 | 92 (4.68) | 32 (1.22) | 3.66 (2.42, 5.54) | <0.001 | 317 (7.00) | 402 (6.64) | 1.19 (1.01, 1.41) | 0.038 |
| Transfer | 434 (3.79) | 132 (5.27) | 1.55 (1.22, 1.95) | <0.001 | 69 (3.51) | 45 (1.72) | 1.94 (1.32, 2.85) | 0.001 | 342 (7.55) | 284 (4.69) | 1.24 (1.05, 1.47) | 0.011 |
| Unknown outcome **^ǂ^** | 608 (8.50) | 132 (5.27) | 1.12 (0.89, 1.41) | 0.336 | 11 (0.56) | 9 (0.34) | 1.52 (0.53, 1.40) | 0.438 | 166 (3.67) | 233 (3.85) | 0.95 (0.76, 1.20) | 0.682 |

*these outcomes include a number of definitions of recovery depending on the individual study protocols. ^ǂ^Children with missing treatment outcomes. ^#^Odds ratio is bivariate association between WAZ < -3 with reference to WAZ ≥-3, adjusted for clustering. SW=severely wasted; MW=moderately wasted.

**Appendix Figure 1: Growth curves for WAZ, disaggregated by WAZ status at admission and wasting type**


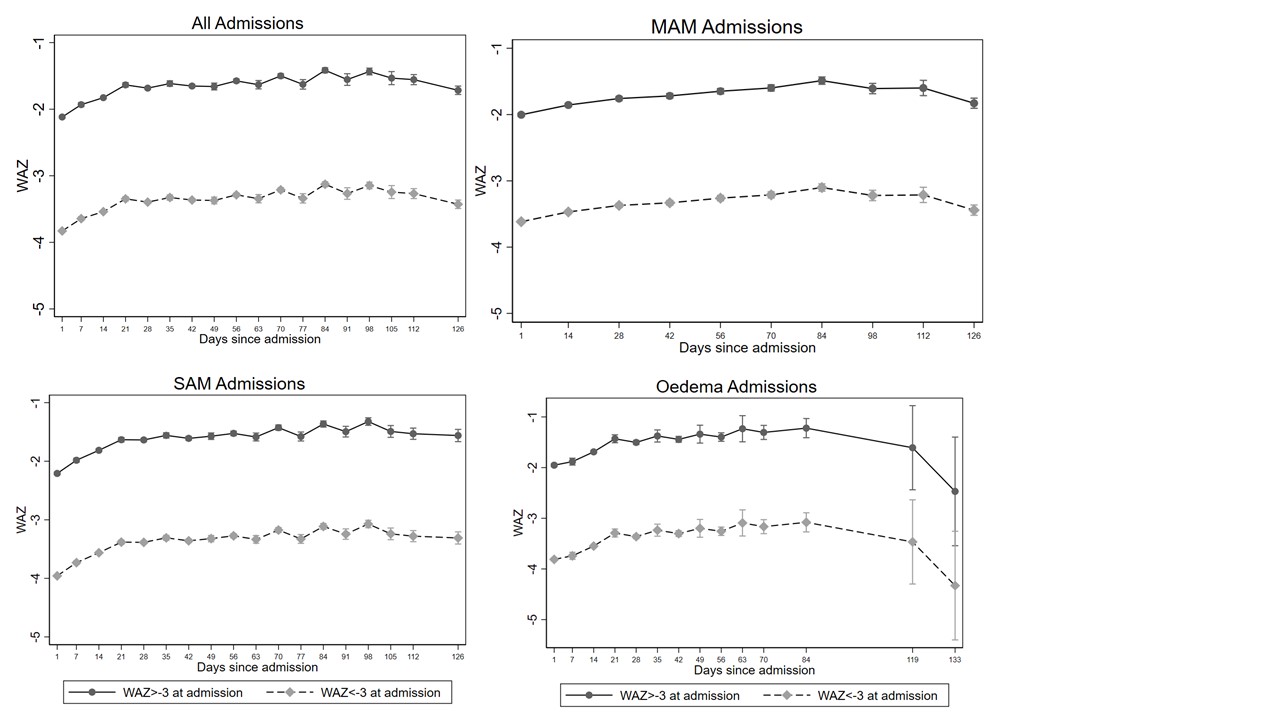


*SAM admission= severe wasting without oedema

**Appendix Figure 2: Growth curves for HAZ, disaggregated by WAZ status at admission and wasting type**


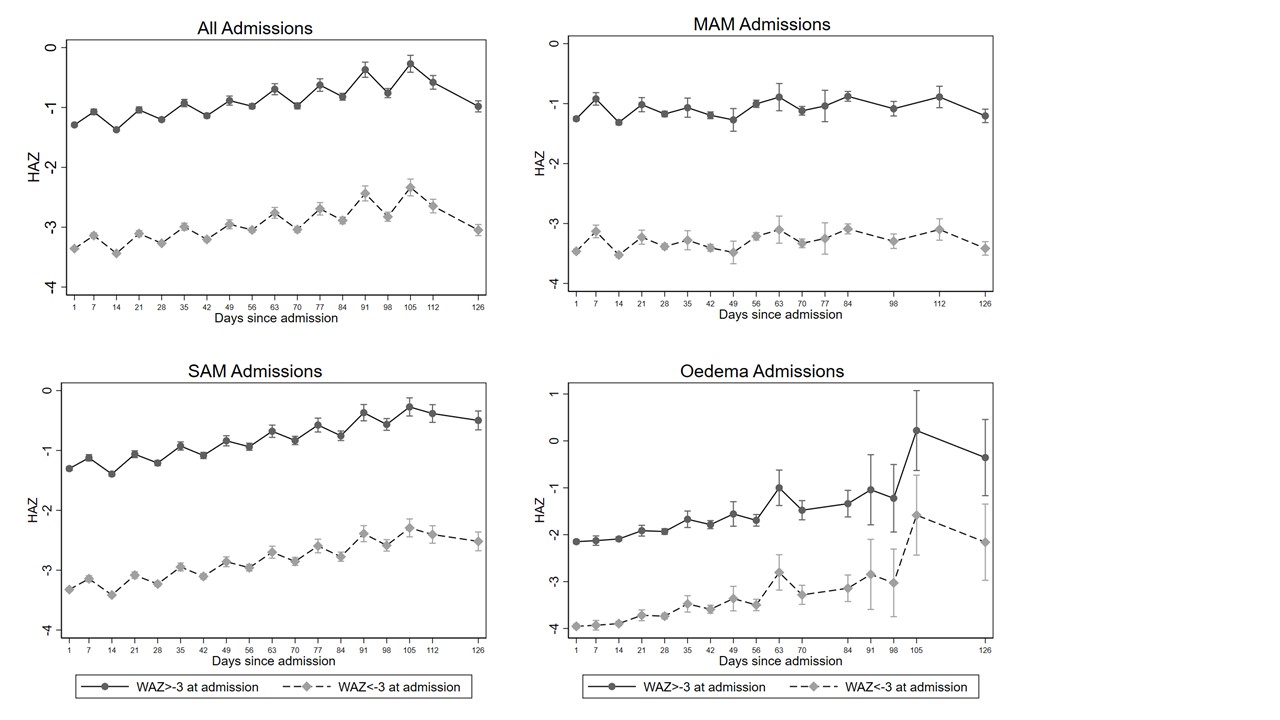


*SAM admission= severe wasting without oedema

**Appendix Figure 3: Growth curves for MUAC, disaggregated by WAZ status at admission and wasting type**


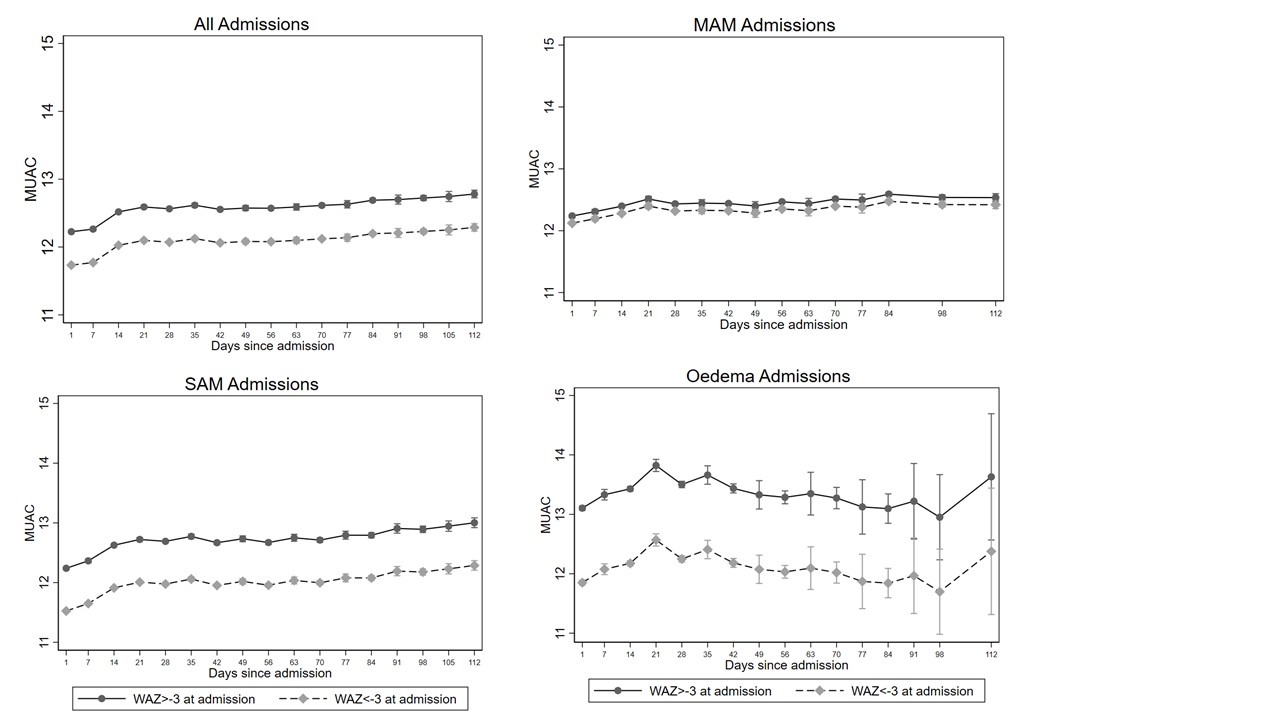


*SAM admission= severe wasting without oedema

**Appendix Figure 4: Growth curves for WHZ, disaggregated by WAZ status at admission and wasting type**


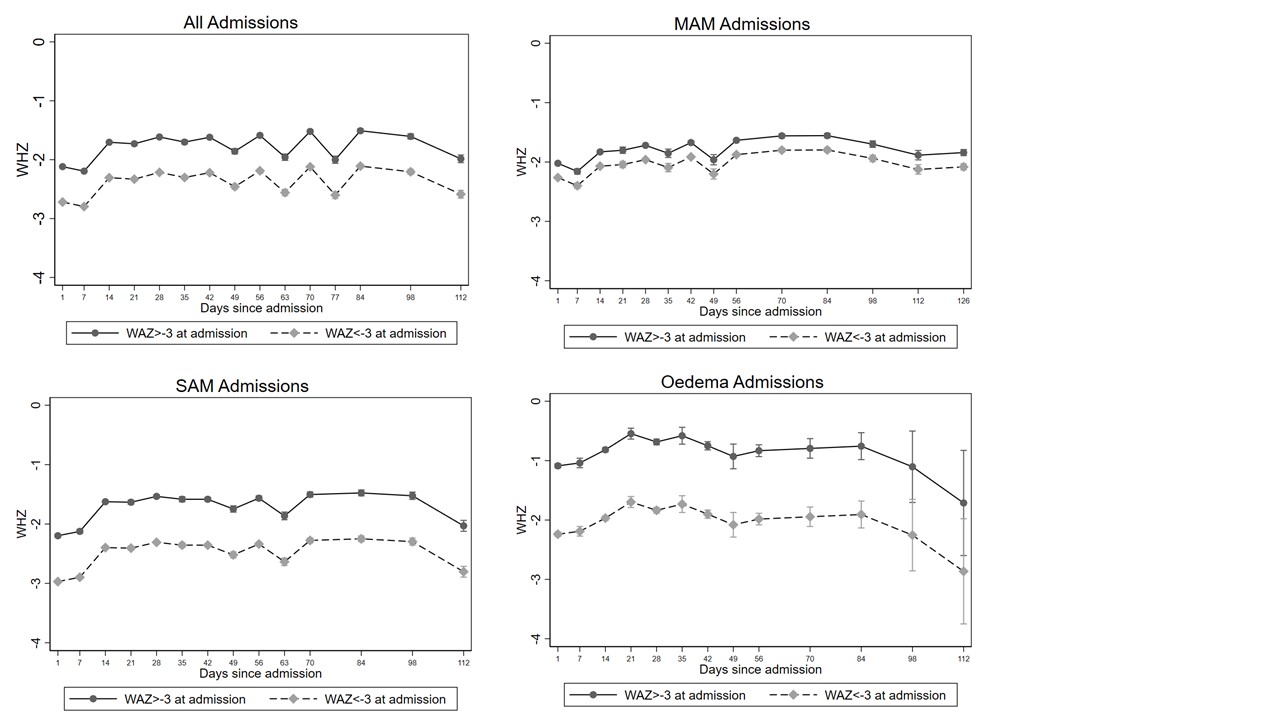


*SAM admission= severe wasting without oedema
